# Curcumin Induces p53-Null Hepatoma Cell Line Hep3B Apoptosis through the AKT-PTEN-FOXO4 Pathway

**DOI:** 10.1155/2017/4063865

**Published:** 2017-07-09

**Authors:** An-Ting Liou, Mei-Fang Chen, Chu-Wen Yang

**Affiliations:** ^1^Department of Microbiology, Soochow University, Shihlin, Taipei 111, Taiwan; ^2^Department of Medical Research, Taipei Veterans General Hospital, Taipei 112, Taiwan

## Abstract

**Objective:**

Curcumin (diferuloylmethane) is a yellow-colored polyphenol with antiproliferative and proapoptotic activities to various types of cancer cells. This study explored the mechanism by which curcumin induces p53-null hepatoma cell apoptosis.

**Results:**

AKT, FOXO1, and FOXO3 proteins were downregulated after curcumin treatment. Conversely, PTEN was upregulated. Subcellular fractionations revealed that the FOXO4 protein translocated from cytosol into the nucleus after curcumin treatment. Overexpression of FOXO4 increases the sensitivity of Hep3B cells to curcumin. Knockdown of the FOXO4 gene by siRNA inhibits the proapoptotic effects of curcumin on Hep3B cell.

**Conclusions:**

This study revealed the AKT/PTEN/FOXO4 pathway as a potential candidate of target for treatment of p53-null liver cancers.

## 1. Introduction

Hepatocellular carcinoma (HCC) is the sixth most common cancer worldwide and is the third most common cause of cancer-related death in the Asia-Pacific region [1]. HCC is a cancer with a high mortality rate [2]. The prognosis of liver cancer, even in developed countries, is very unfavorable. In the US, the five-year survival rates are 5% and 18% before 1989 and after 2005, respectively [3]. It has been reported that the absence of p53 promotes HCC metastasis in a mouse model [4]. Moreover, mutations in the TP53 tumor suppressor gene have been reported in 23–67% of HCC patients worldwide and in 50% of HCC patients in China and South Africa [5].

Curcumin is a natural polyphenol found in the rhizome of* Curcuma longa* (turmeric) [6].* Curcuma longa* has been traditionally used in Asian countries as a medical herb for several pathologies due to its antioxidant, anti-inflammatory [7], cancer chemoprevention [8, 9], and anticancer properties [10–12].

Several studies have reported that curcumin could induce cancer cell apoptosis through p53-dependent and p53-independent pathways [13]. It has been reported that curcumin and related analogous compounds could induce apoptosis in hepatoma cells that express p53 protein normally (e.g., HepG2 cell) through p53/p21 pathway [14–16]. However, little is known regarding the effects of curcumin on p53-null liver cancer cells. In this study, the PI3K/AKT/PTEN/FOXO pathway was shown to mediate curcumin induced apoptosis in p53-null Hep3B hepatoma cells.

## 2. Materials and Methods

### 2.1. Chemicals and Antibodies

Chemicals and PI3K inhibitor LY294002 were purchased from Sigma-Aldrich (Sigma-Aldrich Co. LLC). Antibodies of the signal transduction pathway, apoptosis pathway, and FOXO family were purchased from Cell Signaling (Cell Signaling Technology, Inc.).

### 2.2. Cell Culture

HepG2 (ATCC HB-8065) and Hep3B (ATCC HB 8064), human hepatocellular carcinoma cell lines, were cultured in DMEM (Gibco) containing 10% FBS, 1% NEAA, and 1% glutamine and incubated in an incubator at 37°C with 5% CO_2_.

### 2.3. Flow Cytometry

For cell-cycle analysis, cells were harvested and fixed dropwise with 70% ethanol. After incubation overnight at 4°C, ethanol was removed by centrifuge, and RNase A was added, followed by a propidium iodide (PI) solution. The cell-cycle stages of stained cells were analyzed by flow cytometry (Cytomics FC500, Beckman). For caspase analysis, 25 *μ*L of FITC labeled caspase substrates (OncoImmuno, Inc.) was incubated with cells for 45 mins. PI solution was added and incubated for another 15 mins. Caspase substrate and propidium iodide were removed by centrifuge and cells were washed by PBS. Caspase intensity was detected by flow cytometry.

### 2.4. Immunoblotting

Each well of polyacrylamide gel was loaded with 55 *μ*g cell lysate. After SDS-PAGE, the stacking gel was discarded and separating gel was taken to the semi-dryer blotting system. Once blotting was finished, the nitrocellulose (NC) membrane was washed with TTBS and was blocked by 5% milk in TTBS solution. Washed NC membrane was incubated with specific primary antibodies with shaking at 4°C overnight. Secondary antibody solution was added and incubated with shaking at room temperature for 1 hour. NC membrane with secondary antibody was washed with TTBS 3 times to remove nonspecific binding. Enhanced chemiluminescence (ECL) substrate solution was added to determine the results using luminescence readers.

### 2.5. Cloning and Sequencing

PCR primers of FOXO4 gene were designed by the Primer 3 software (http://bioinfo.ut.ee/primer3-0.4.0/primer3/). The forward primer sequence is FOXO4F 5′-GAGCAGGAAGCTGAGTGAGAG-3′. The reverse primer sequence is FOXO4R 5′-CCTAGTCCCAGGACGCTAGTC-3′. PCR products were subcloned into pcDNA 3.1 TOPO TA Expression Vector (Invitrogen) and validated by Sanger sequencing in the Genome Center of National Yang-Ming University.

### 2.6. RNA Interference

siRNAs of PTEN, FOXO1, FOXO3a, and FOXO4 genes were purchased from Invitrogen. siRNAs were transfected using Lipofectamine 2000 (Invitrogen) according to the instructions of the manufacturer. The effectiveness of the siRNAs on protein expression was validated by Western blotting.

### 2.7. Statistics

Nonparametric two sample tests were performed using the Mann-Whitney *U* test by the wilcox.test function of R. *p* values less than 0.05 were considered to be statistically significant.

## 3. Results

### 3.1. HepG2 and Hep3B Cells Exhibit Different Sensitivity in Curcumin Induced Cell Death

**Figure 1:**
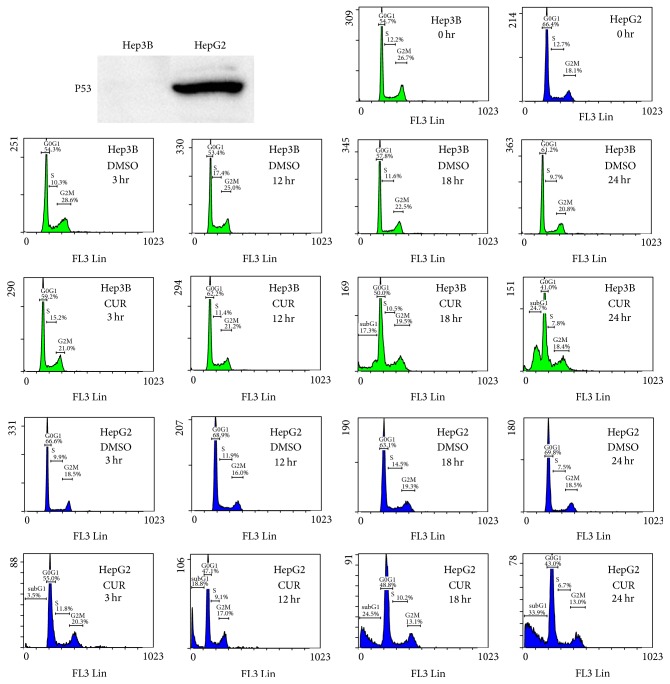
Curcumin treatments induces HepG2 and Hep3B cell death. The concentration of curcumin used in the experiments was 50 *μ*M. The data are representative of triplicate experiments. CUR: curcumin. DMSO: dimethyl sulfoxide (solvent of curcumin). *x*-axis indicates propidium iodide (PI) intensities.

The p53 protein expression in HepG2 and Hep3B cells was first checked by Western blot analysis (Figure 1). As expected, p53 protein is expressed in HepG2 cells but not expressed in Hep3B cells. Flow cytometry was used to analyze curcumin induced HepG2 and Hep3B cell death (Figure 1). The subG1 portions of HepG2 cells were increased from 18.8 ± 1.5%, 24.5 ± 3.6%, and 33.9 ± 2.9% after 50 *μ*M curcumin treatment at 12, 18, and 24 hours, respectively. In contrast, the subG1 portions of Hep3B cells were 17.3 ± 1.3% and 24.7 ± 3.1% after 50 *μ*M curcumin treatment at 18 to 24 hours, respectively. These results suggested that HepG2 cells (which express wild-type p53 protein) were more sensitive to curcumin treatment than p53-null Hep3B cells.

### 3.2. Curcumin Induces Apoptosis in Hep3B Cells

**Figure 2:**
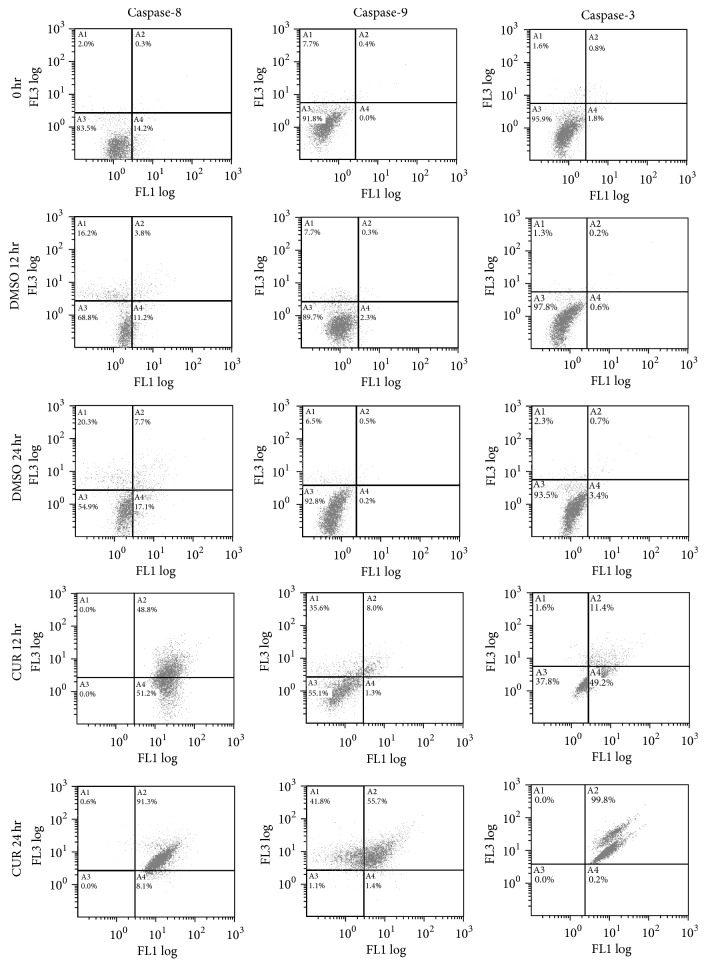
Flow cytometry analysis for caspase activity after curcumin and DMSO treatments in Hep3B cells. The concentration of curcumin used in the experiments was 50 *μ*M. *y*-axis indicates propidium iodide (PI) intensities; *x*-axis indicates caspase activities. The data are representative of triplicate experiments. CUR: curcumin. DMSO: dimethyl sulfoxide (solvent of curcumin).

**Figure 3:**
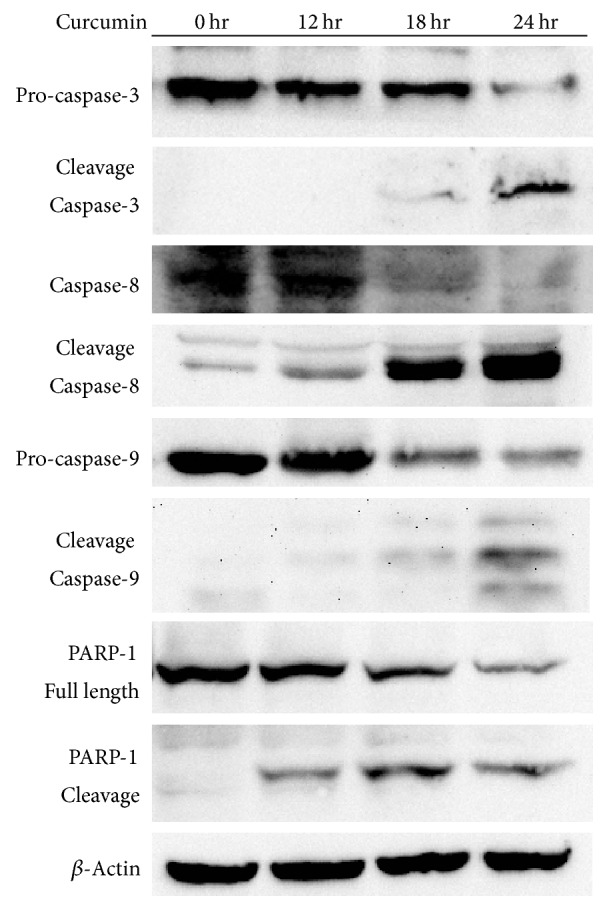
Western blot analysis for caspases and PARP-1 cleavages after curcumin treatments in Hep3B cells. The concentration of curcumin used in the experiments was 50 *μ*M. The data are representative of triplicate experiments.

To confirm that the subG1 portion observed in Hep3B cells shown in Figure 1 was due to apoptosis, caspase-3, caspase-8, and caspase-9 activities were analyzed by flow cytometry. As shown in Figure 2, caspase-3, caspase-8, and caspase-9 were activated after 12 hours of curcumin treatments. Western blotting further confirmed the results of flow cytometry assays (Figure 3). The observations that both caspase-8 and caspase-9 were activated indicate that curcumin induced apoptosis may be mediated by both of the extrinsic and intrinsic pathways simultaneously.

### 3.3. Treatments of Curcumin Activate AKT/PTEN/FOXO Pathway in Hep3B Cells

**Figure 4:**
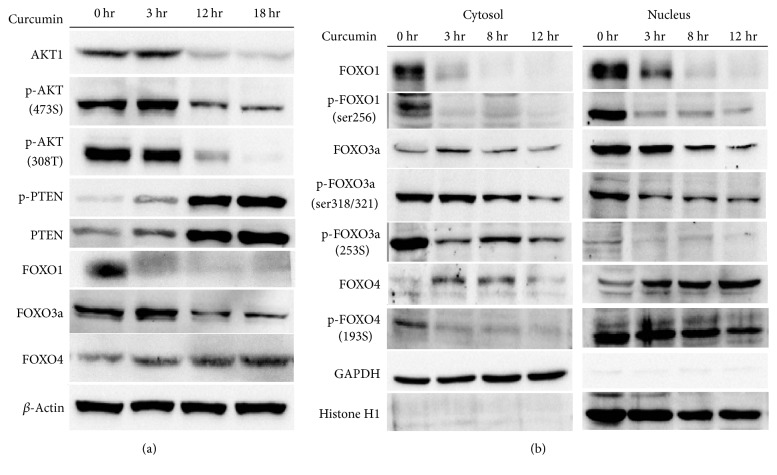
Curcumin treatments induce AKT pathway in Hep3B cells. (a) Western blot analysis for AKT activation after curcumin treatments. (b) Western blot analysis for FOXO protein localization after curcumin treatments in Hep3B cells. The concentration of curcumin used in the experiments was 50 *μ*M. The data are representative of triplicate experiments.

Since the Hep3B cells are p53-null, the p53/p21 pathway for apoptosis is defective; the response of AKT/PTEN pathway (for cell survival signal) to curcumin treatment was analyzed by Western blot analysis. As shown in Figure 4(a), PTEN and phospho-PTEN proteins increased accompanied by the decreased native and phosphorylated forms (p-AKT 308T and 473S) of the AKT1 protein after treatments of 50 *μ*M curcumin. The FOXO1 and FOXO3a proteins were downregulated after 50 *μ*M curcumin treatments (Figure 4(a)). In contrast, the FOXO4 protein was upregulated after 50 *μ*M curcumin treatments. Subcellular fractionation revealed that the FOXO1 and FOXO3a proteins were downregulated in both cytosol and the nucleus after 50 *μ*M curcumin treatments (Figure 4(b)). On the other hand, the FOXO4 protein was translocated from cytosol to the nucleus.

### 3.4. PI3K Inhibitor Enhances Curcumin Induced Caspase 3 Activation in Hep3B Cells

**Figure 5:**
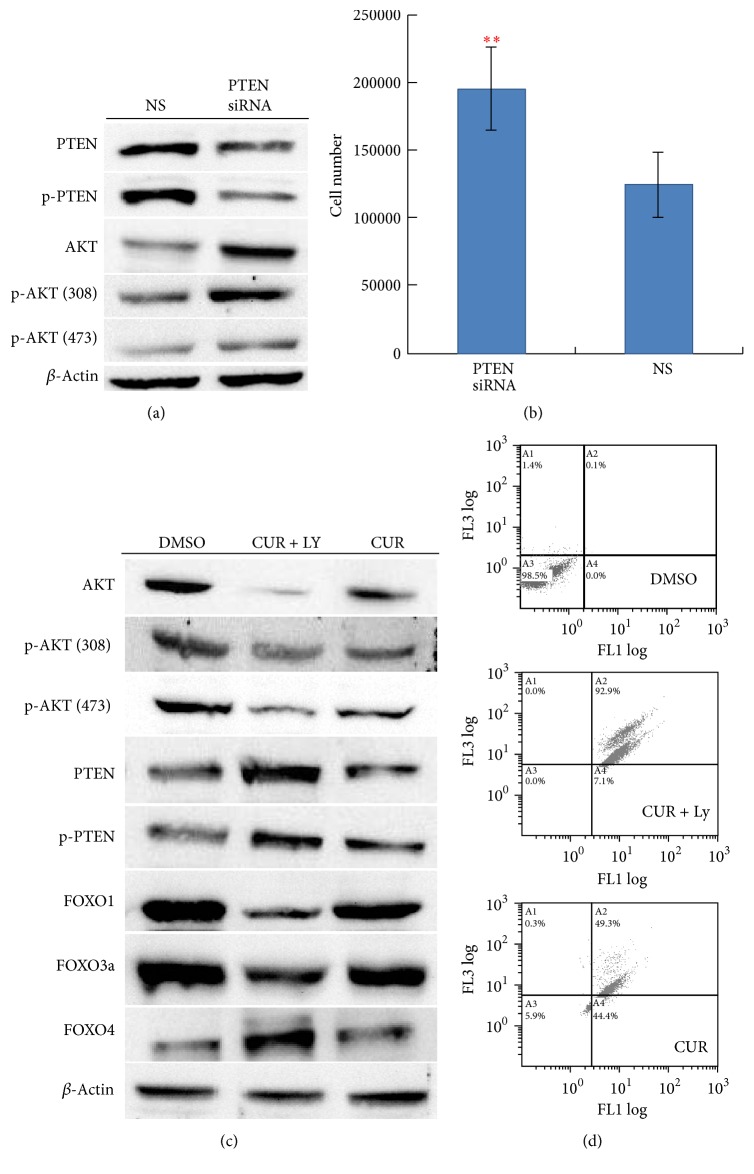
Associations of PI3K/AKT1/PTEN and curcumin treatment in Hep3B cells. (a) Western blot analysis for AKT1 activation after PTEN knockdown. (b) Hep3B cell viability after PTEN knockdown. The “*∗∗*” indicates Mann-Whitney *U* test, *p* < 0.01. (c) Western blot analysis for AKT1/PTEN/FOXOs protein expression in experiments with and without addition of PI3K inhibitor LY294002 before curcumin treatments. (d) Flow cytometry analysis for caspase-3 activities in experiments with and without addition of PI3K inhibitor LY294002 before curcumin treatments. *y*-axis indicates propidium iodide (PI) intensities; *x*-axis indicates caspase activities. The concentration of curcumin used in the experiments was 50 *μ*M. The data are representative of triplicate experiments. CUR: curcumin. DMSO: dimethyl sulfoxide (solvent of curcumin). LY: LY294002.

Knockdown of PTEN by siRNA in Hep3B cells led to an increase in the native and phosphorylation forms (p-AKT 308T and 473S) of AKT1 proteins (Figure 5(a)) and an increase in cell growth (Figure 5(b)). To elucidate whether the downregulation of phospho-AKT1 proteins and upregulation of PTEN proteins are associated with PI3K activity, the PI3K inhibitor LY294002 was used to treat Hep3B cells with curcumin. As shown in Figure 5(c), the effects of curcumin on AKT1 and PTEN were enhanced by addition of the PI3K inhibitor LY294002. Flow cytometry assays indicated that the caspase-3 activity induced by curcumin was repressed by addition of the PI3K inhibitor LY294002 (Figure 5(d)). These results suggest that PI3K activity might be associated with curcumin induced upregulation of PTEN proteins and downregulation of native and phospho-AKT1 proteins in Hep3B cells.

### 3.5. FOXO4 Knockdown Reduces Curcumin Induced Apoptosis in Hep3B Cells

**Figure 6:**
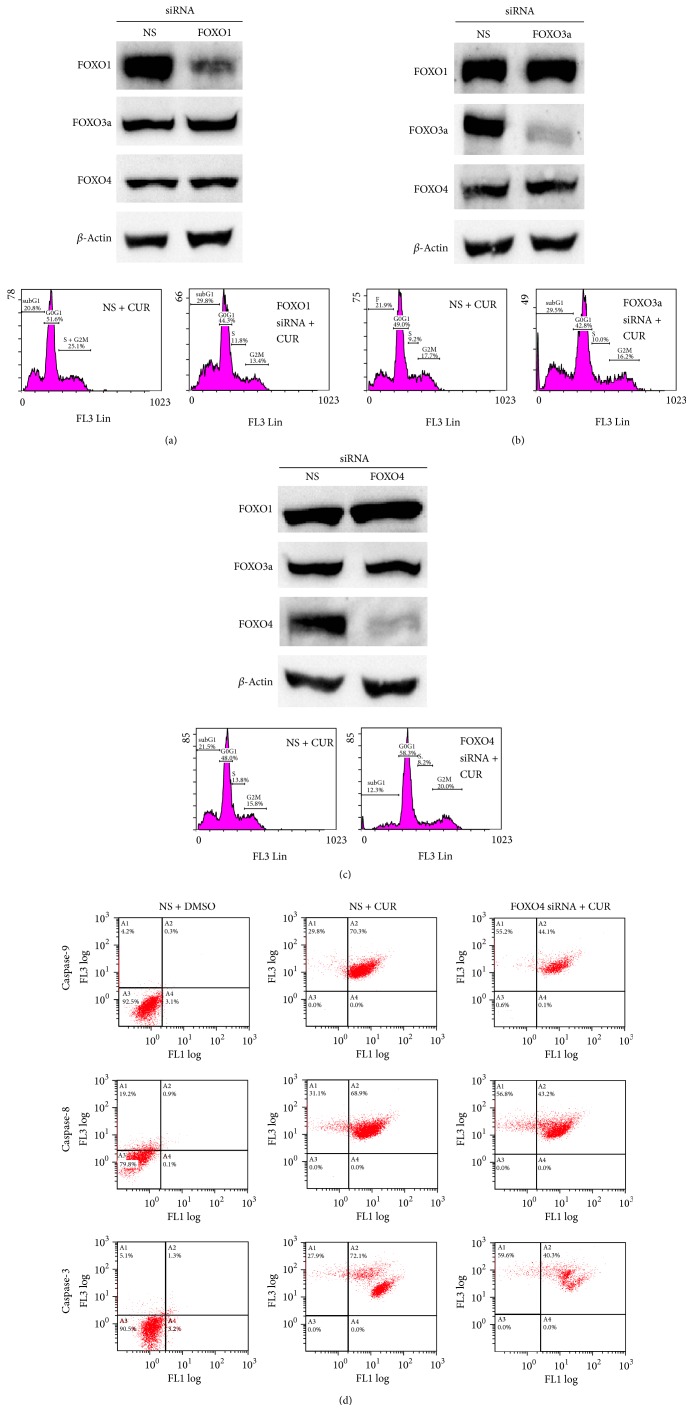
Effects of FOXO knockdown on curcumin induced Hep3B cell apoptosis. (a) FOXO1 knockdown; (b) FOXO3a knockdown; (c) FOXO4 knockdown; (d) caspase activities of FOXO4 knockdown experiments. *y*-axis indicates propidium iodide (PI) intensities; *x*-axis indicates caspase activities. The concentration of curcumin used in the experiments was 50 *μ*M. The data are representative of triplicate experiments. CUR: curcumin. DMSO: dimethyl sulfoxide (solvent of curcumin).

To reveal the roles of FOXO family proteins in curcumin induced Hep3B cell apoptosis, specific siRNAs were used to knockdown FOXO1, FOXO3a, and FOXO4. As shown in Figures 6(a) and 6(b), knockdown of FOXO1 and FOXO3a slightly enhances the curcumin induced Hep3B cell apoptosis. However, knockdown of FOXO4 reduced subG1 portion of Hep3B cell after curcumin treatments (Figure 6(c)). Flow cytometry assays indicated that the caspase-8, caspase-9, and caspase-3 activities induced by curcumin were reduced by knockdown of FOXO4 (Figure 6(d)). These results suggest the role for FOXO4 protein in mediating curcumin induced Hep3B cell apoptosis.

### 3.6. FOXO4 Overexpression Increases Curcumin Induced Apoptosis in Hep3B Cells

**Figure 7:**
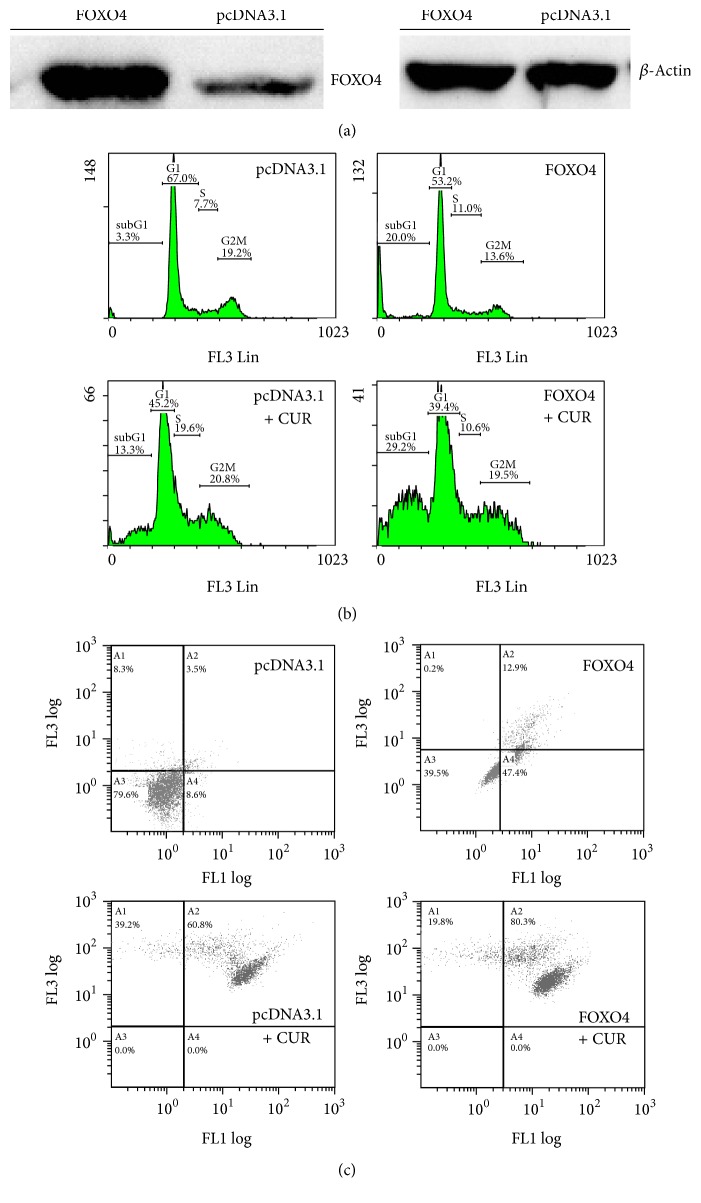
Effects of FOXO4 overexpression on curcumin induced Hep3B cell apoptosis. (a) Western blot analysis of overexpressed and endogenous FOXO4 proteins in Hep3B cells. (b) Effects of FOXO4 overexpression on Hep3B cell apoptosis with and without curcumin treatments. (c) Caspase-3 activities in FOXO4 overexpression experiments. *y*-axis indicates propidium iodide (PI) intensities; *x*-axis indicates caspase activities. pcDNA 3.1 is a mammalian expression vector with the CMV promoter (Invitrogen). The concentration of curcumin used in the experiments was 50 *μ*M. The data are representative of triplicate experiments. CUR: curcumin. DMSO: dimethyl sulfoxide (solvent of curcumin).

To confirm the role of the FOXO4 protein in curcumin-induce Hep3B cell apoptosis, the* FOXO4* gene was cloned and overexpressed in Hep3B cells (Figure 7(a)). As shown in Figures 7(b) and 7(c), overexpression of FOXO4 in Hep3B cells induced apoptosis. Moreover, Hep3B cells with overexpressed FOXO4 proteins exhibited higher sensitivity to curcumin.

## 4. Discussion

The expansion of tumor cell populations is dependent on both the rates of cell proliferation and cell death. Apoptosis is a major source of cell death. Therefore, agents that trigger apoptosis/cell death could be the most promising candidates of cancer therapies. In the past years, a number of studies have been published describing the anticancer effects of curcumin. These investigations could be divided into three categories: (1) anti-inflammatory, antioxidant, and chemoprevention activities [8, 9]; (2) antiproliferative and proapoptotic activities [11–13]; (3) anti-invasive and antimetastasis activities [10]. It has been shown that curcumin modulates various targets including inflammatory pathway, transcription factors, protein kinases, the cell survival pathway, drug resistance proteins, adhesion molecules, growth factor receptors, and the cell-cycle regulatory pathway [6]. One of the major pathways regarding antiproliferative and proapoptotic activities of curcumin is the p53-dependent (p53/p21) pathway [13]. As shown in Figure 1, the HepG2 hepatoma cell that expresses wild-type p53 protein is more sensitive to curcumin induced cell death than the p53-null Hep3B cell. On the other hand, p53-independent pathways such as the PI3K/PTEN/AKT pathway, stress response pathways, and the NF-kB pathway were also found to be activated by curcumin to induce cancer cell death [11]. In this study, the PI3K/PTEN/AKT/FOXO pathway was shown to be activated to induce p53-null Hep3B hepatoma cell apoptosis after curcumin treatment.

Treatment with curcumin inhibits the PI3K/AKT pathway in various types of cancer cells including breast cancer [17], lung cancer [18], colon cancer [19], renal cancer [20], ovarian carcinoma [21], pancreatic cancer [22], osteosarcoma [23], acute T cell leukemias [24], and liver cancer [25]. The results of this study are consistent with these reports. However, the consequence of PI3K/AKT pathway inhibition is different among different types of cancer cells. For example, Zhao et al. demonstrated that curcumin inhibits the PI3K/AKT pathway and induces FOXO1 expression in pancreatic cancer cells [22]. In contrast, inhibition of the PI3K/AKT pathway and induction of FOXO4 in curcumin treated p53-null hepatoma cell were observed in this study. The diverse results among studies may be due to several reasons. The first possible reason is the tissue specificity of gene (pathway) function. Different cells (in different tissues) share common core signal transduction pathways with different downstream effectors. As a consequence, activation of a common core signal transduction pathway leads to different responses in different type of cells. Second, different genetic backgrounds among cancer cells may cause different responses to curcumin. As shown in Figure 1, the HepG2 cell which expresses wild-type p53 protein exhibits different sensitivity to curcumin compared with the p53-null Hep3B cell. Third, the signal transduction pathways in cells are networks that perform crosstalk with and influence each other. However, usually only one or two major pathways are focused and observed in one study. Therefore, a very limited scope is typically obtained in one study. In this study, the FOXO4 miRNA could not completely reverse the effects of curcumin treatment. It has been shown that curcumin has multiple targets in the signal transduction pathway network [6–12]. Therefore, the results in this study could not rule out that there are other pathways which also respond to curcumin in Hep3B cells.

Yang et al. have shown that FOXO4 overexpression inhibits AKT activity, increases p27 Kip1 transcription, and suppresses HER2-overexpressed tumors [26]. Zhang et al. showed that upregulation of cyclin-dependent kinase inhibitors, p21 (Cip1) and p27 (Kip1), enhanced transactivation of FOXO4 factor and dysregulation of AKT signaling associated with inhibition of cell proliferation and tumorigenicity of hepatoma cells [27]. Xie et al. have shown that inhibiting the PI3K/AKT signaling pathway enhanced the transcriptional activity of FOXO4 associated with upregulation of the cyclin-dependent kinase (CDK) inhibitors p21Cip1 and p27Kip1 and reduced proliferation of breast cancer cells [28]. Roy et al. showed that inhibition of the PI3K/AKT pathway and activation of FOXO4 transcriptional activity led to cell-cycle arrest and apoptosis in pancreatic cancer cells [29]. In contrast, Chen et al. have shown that downregulation of FOXO4 by miR-421 induces cell proliferation and apoptosis resistance in human nasopharyngeal carcinoma [30]. Li et al. have shown that targeting FOXO4 by miRNA-150 promotes cervical cancer cell growth and survival [31]. These studies are consistent with results of this study; namely, FOXO4 has antiproliferative and proapoptotic activities. Moreover, Yang et al. showed that FOXO4 sensitized cells to apoptosis induced by the chemotherapeutic agent 2-methoxyestradiol [26]. A similar phenomenon was observed in this study as overexpression of FOXO4 in Hep3B cells increased sensitivity to curcumin.

## 5. Conclusions

**Figure 8:**
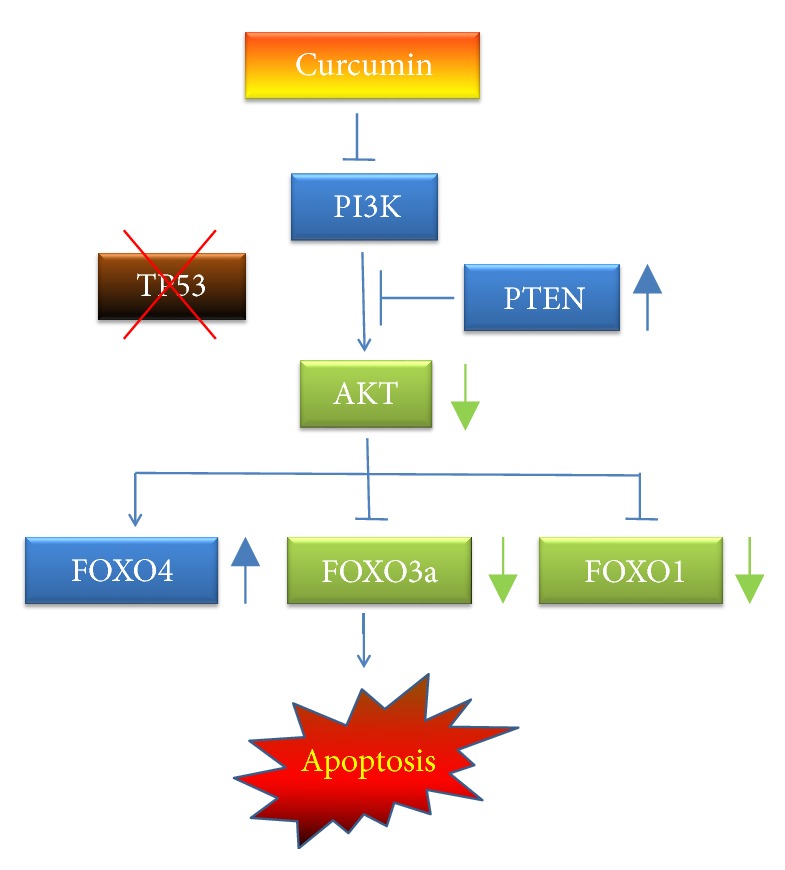
A schematic model describing the mechanism by which curcumin induces Hep3B cell apoptosis via the PI3K/AKT/FOXO pathway.

A model, as shown in Figure 8, is proposed according to results of this study. In p53-null Hep3B cells, treatment with curcumin upregulates PTEN proteins and downregulates AKT1 proteins. As a consequence, translocation of the FOXO4 protein into the nucleus leads to Hep3B cell apoptosis. The results of this study revealed the AKT/PTEN/FOXO4 pathway as a potential candidate target for treatment of p53-null liver cancers.
